# Fostering ethical reflection on health data research through co-design: A pilot study

**DOI:** 10.1007/s40889-022-00148-4

**Published:** 2022-06-22

**Authors:** Joanna Sleigh, Julia Amann

**Affiliations:** Health Ethics and Policy Lab, Swiss Federal Institute of Technology (ETH Zurich), Zurich, Switzerland

**Keywords:** Co-design, Bioethics, Ethical principles, Visualisation, Ethics education, Research ethics

## Abstract

Health research ethics training is highly variable, with some researchers receiving little to none, which is why ethical frameworks represent critical tools for ethical deliberation and guiding responsible practice. However, these documents' voluntary and abstract nature can leave health researchers seeking more operationalised guidance, such as in the form of checklists, even though this approach does not support reflection on the meaning of principles nor their implications. In search of more reflective and participatory practices in a pandemic context with distance learning, this study explored whether co-design could support engagement and reflection on ethical principles amongst early-stage health researchers. In a pilot test using the Swiss Personalized Health Network's ethical framework for Responsible Usage of Personal Data in Health Research as a case study, we engaged health researchers to design visuals of four ethical principles. In two online workshops, participants (N = 10, N = 8) completed activities such as individual reflection, collaborative ideation, sketching, prototyping, discussion, and feedback. Our analysis shows that the co-design process helped foster in-depth engagement and reflection on the meaning and relevance of the SPHN ethical principles. Participants reported enjoying the experience, and most felt that visuals could motivate engagement with ethical frameworks. This study demonstrates the feasibility of a participatory, design-oriented approach to promote engagement with research ethics among early-career health researchers and highlights key challenges and lessons learned. In doing so, it lays the foundation for future research to investigate the impact of design-oriented, participatory learning to foster reflection and deliberation in ethics education.

**Supplementary Information:**

The online version contains supplementary material available at 10.1007/s40889-022-00148-4.

## Background

In today’s data-driven health ecosystem, stimulated by a pandemic and tarnished with data breaches and privacy scandals, ethical guidelines and frameworks represent important tools to promote good data management practice and responsible innovation (Martin et al. 2019). A policy landscape analysis of data sharing guidelines using digital bioethics methods exemplifies how hundreds of such documents exist, as every research institute and organisation working with digital technologies publishes policy tools to support compliance, ethical reflection and engender trust (Blasimme et al. 2018; Schneider et al. 2021). Although diverse in format, style, focus, and underlying moral theories, these bioethics artefacts have a common goal: to communicate normative indications and principles to support ethical decision-making. More specifically, they educate and sensitise readers on ethical practice, act as a means of deterrence and discipline, and provide users with a formal document to turn to when in conflict. For example, researchers working with biomedical data within the health sector use these documents (notably their checklists) when planning and reporting projects to research ethics committees.

Despite taking a central place in ethics policy and education, ethical frameworks and guidelines face adoption challenges. For one, they are voluntary, and second, they communicate knowledge that is often abstract, highly conceptual, and tied to tacit knowledge, which many researchers lack, especially those still in training (Forsström et al. 2017). Adding to the challenge is that their audience comprises experts and non-experts, and due to limited resources they often remain in linear verbo-centric formats with little user testing nor attention to user needs or preferences (Giacomini et al. 2009; Sleigh et al. 2020). Resultantly, we increasingly see checklists incorporated to operationalise these framework’s soft and informal guidance and thereby help practitioners make ethical decisions (Paeffge et al. 2021). However, if used as a checklist, these tools can easily be misused and may thus fail to provoke ethical reflection and reasoning as critical and transferrable skills (Madaio et al. 2020). So what can we do to support early-career health researchers’ reflection on ethical frameworks, particularly during a pandemic setting with distance learning and in a university context where ethics education is often limited (Beever et al. 2021).

One potential approach to promote early-career health researchers’ engagement with ethical frameworks is visuality. Although other fields such as mathematics and science have recognised the pedagogic benefits of using visualisation to engage learners (Mickelson and Ju 2010), visuality in bioethics education remains limited to visual education tools such as comic strips and films (Ike and Anderson 2018). For early-stage researchers in a university context, the primary tools for learning ethics are rather ethical dilemmas and cases, discussion in peer groups, and collections of online learning materials (Tammeleht et al. 2022). However, the integration of visuality holds potential for immediate engagement, for visuals can promote “awareness of ethical problems that not only affect the minds of people, but they engage their appetites, beliefs, emotions and desires as well” (Solbakk 2015).

Visuality’s benefits within a learning context can further be enhanced by participatory practices (Literat 2012). Here, we refer to approaches such as co-design, co-production, design thinking and design jams which have evolved over the years and are informed by various political, pragmatic and theoretical arguments (Liegl et al. 2015; Törpel et al. 2009). In addition, Science and Technology Studies (STS) provides various theoretical resources concerning: participatory practices, the emergent nature and diversity of publics, and the need for 'democratizing technological culture' (Bijker 2003). Moreover, scholars recommend stakeholder engagement and meaningful participation to foster trust, accountability, and more inclusive public policy and data-sharing governance (Blomkamp 2018; Goodyear-Smith et al. 2015; Kaye et al. 2018). Yet, although the language and activities of design and participation are today widely used, their application to ethical frameworks rarely occurs.

As co-design applied to ethics education is rarely described in the literature, this study aimed to explore whether co-design can support engagement and reflection on ethical principles amongst early-stage health researchers. With this goal we used the Swiss Personalized Health Network's (SPHN) ethical framework for the Responsible Usage of Personal Data in Health Research (SPHN 2018) as a case study, with focus on its’ four principles Respect of Persons, Privacy, Accountability, and Data Fairness.

## Methods

### Design & procedure

**Fig. 1:**
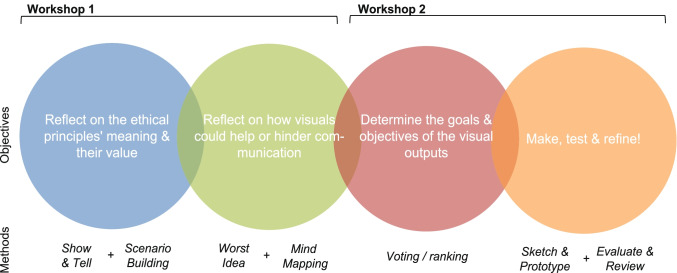
Co-design workshop methods and objectives. Circles indicate the four objectives with the methods used to achieve each objective indicated by labels

In October 2021, we conducted two online co-design workshops following a co-creation approach similar to Forsström et al. (2017), emphasising collectively creating visible, tangible referents to explain and concretise ideas. Specifically, participants were invited to co-design visuals for the four ethical principles of the SPHN framework: Respect of Persons, Privacy, Accountability, and Data Fairness. In doing so, the co-design workshops aimed to foster ethical reflection and deliberation among participants. The workshops were led by the principal investigator, a visual anthropologist experienced in graphic design, alongside a health communication researcher experienced in co-design facilitation. Informed by co-design and co-production literature (Iversen et al. 2010; Sanders and Stappers 2008; Sbaiti et al. 2021), we incorporated participants into various phases of the design process as those most directly affected by a design outcome should have a substantive say in what that outcome is. The activities selected were drawn from participatory design literature (Mastrianni et al. 2021; Williams and Tembo 2021) and harnessed different communication modes, including individual reflection, collaborative ideation and discussion, alongside making and testing prototypes. Figure 1 illustrates the activities and their relative objectives, while details of each activity are provided below. We also followed the INVVOLVE framework’s recommendations for practice (Slattery et al. 2020).

#### Pre-workshop activity

Before the workshops, we asked participants to complete a preparation activity using the online tool Miro.com. In this way, they practised using the digital whiteboard tool and became familiarised with its functionalities. This activity also asked participants some background questions such as how many years they had worked with data, what type of health data they worked with, what the last framework they used was, what was the context of use, and generally how frequently they engaged with ethical guidelines. We also provided participants with a link to the SPHN ethical framework and asked them to represent their reading experience using both an image and text for reflection and creative thinking.

#### Workshop 1

The first co-design workshop opened with an ice-breaker activity titled ‘Show & Tell’ wherein participants introduced themselves and explained their visual representation of the reading experience. Following, was a scenario-building activity with the goal to reflect on the principles’ meaning and their value in research practice. This scenario method comes not only from co-design and future and foresight research (Jessen et al. 2018; Wright et al. 2020), but also from the fields of bioethics and public health ethics, where it helps to build the capacity of moral and ethical awareness (Schröder-Bäck et al. 2014). With participants divided into groups, the task entailed evaluating each principle’s description and using digital sticky notes to brainstorm real-world examples that concisely demonstrated the issues and ramifications the principles address. The task requested attention to the context (where story took place), the stakeholders (who was involved), the event (what occurred and how), the reasons (why it occurred), alongside the consequences and who was impacted (what consequences for whom). In the second half of the workshop, participants collaborated in mapping out how they felt visuals could help or hinder engaging with ethical principles. This mind-mapping activity is frequently used in design-thinking (Kokotovich 2008) and contributed to a shared discussion of objectives and limitations of visuals in communicating complex information.

#### Workshop 2

The second co-design workshop sought to further explore the visualisation of ethical concepts by engaging participants in discussing the nature of referents (Pauwels 2021), collectively defining design criteria, alongside ideating visible and tangible referents that would explain and concretise each principle. The workshop began with an outline of objectives and a recap of the previous workshop, thereby providing participants with the opportunity to add, change or comment on the results and findings already drawn. The first activity was ‘Worst Idea Possible’, wherein participants had two minutes to write or sketch the worst imaginable visual of these principles. After this, they had to turn someone else’s bad idea into something good. This creative tool of reverse thinking sought to relax participants whilst boosting their creativity and confidence (Birsel 2017; Dam and Siang 2021). After this activity, participants voted on their preferred design criteria and visual characteristics using typologies from visual communication literature (Pauwels 2021; Saunders 1994). Voting categories included graphic type, engagement style, level of detail, representational convention, use of story, colour, text, and overall style, with subcategories listed in supplementary file 1. Participants spent the rest of the workshop conducting rapid sketching and discussion, methods traditionally used in co-design workshops to promote collaboration and engagement of all stakeholders (Southwick et al. 2021). In this last stage, we encouraged participants to visualise their idea by sketching using paper and pen and then showing their sketch to the group on the screen or using post-it notes. As well, we asked them to explain their visuals during the discussion. The workshop concluded with participants choosing how to continue their involvement – as a decision-maker, creator, or no more involvement – and completing a voluntary evaluation survey comprising 15 questions of Likert-type scales and open comments. The survey took approximately 10 min to complete.

#### Post-workshops

After the workshop, participants continued collaborating in their self-selected roles as either designers or decision-makers with the end goal to transform the prototypes into finished products. Activities in the designer role involved ideating, sketching, and prototyping the visualisations, whilst decision-makers provided feedback after each design session. We documented this feedback in open text comments and a five-point Likert scale survey based on criteria drawn from the voting and workshop discussions. As well, a knowledge visualisation expert provided feedback in a final evaluation. This process resulted in four visuals which the collective approved, and these are presented in the results section.

### Participants

For this pilot-test, we recruited health researchers as they are the primary target group of the SPHN Ethical Framework, specifically those working in a biomedical field and using primary or secondary health data. These researchers would ideally have received SPHN funding or be working on SPHN related projects. Recruitment of participants with this background took a three-phased approach. First, an open call-out was shared with researchers who had received SPHN funding and female members of their groups (to ensure gender balance in outreach). Second, the call-out was distributed amongst relevant research groups and labs, and third, it was published by relevant university groups, such as journal clubs and associations. Interested participants filled out an online registration form. To be included in the study, participants had to be working with health data, speak English and be willing to co-design an output. Participation was voluntary, with written informed consent obtained.

Ten participants took part in the first co-design workshop, of which eight partook in the second. These individuals studied or worked at the university. Table 1 presents an overview of the participants’ characteristics.

**Table 1:** Demographics of participants: Gender, education, number of years working with data, and reported frequency of engagement with ethical framework

Category	N
Gender	
Male	4
Female	6
Education	
Pre-graduate	6
Post-graduate	4
Years working with data	
< 2	5
3–4	3
5 >	2
Frequency of engagement with ethical frameworks	
Unsure	1
Once	1
Sometimes	5
No response	2

### Data collection

To document participant engagement and ethical reflection during the workshops, we collected data in various ways about what participants did, said, and made. The preparation activity responses were documented on Miro.com, with screenshots taken to capture the results. The online co-design workshops were recorded using the video conferencing tool Zoom. Participants’ ideas and preferences were also recorded on the Miro.com digital whiteboard platform using digital sticky notes, text comments, and drawings/sketches. As well, each of the facilitators individually made field notes both during and immediately after the co-design sessions. The focus of these notes was to document aspects not recorded in Miro.com, such as group dynamics, difficulties or challenges of the tasks, and the processes of idea generation. An overview of the data material is presented in Table 2.

**Table 2:** Overview of data material: details of the data types collected during each activity and analysed in this study

Data collection tool / activity	Data included in the analysis
Survey	Survey responses
Show and tell	Pictures (Miro.com screenshots) and oral description of visual by participant during WS.1 (zoom recording)
Scenario-building	Pictures (Miro.com screenshots) and oral description of visual by participant during WS.1 (zoom recording)
Mind-Mapping	Picture (Miro.com screenshot) and oral discussion during WS.2 (zoom recording)
Voting exercises	Picture (Miro.com screenshot) and oral discussion during WS.2 (zoom recording)
Sketching	Drawings, notes, and Adobe InDesign drafts
Review process	Survey responses and participant comments
Descriptive and interpretative fieldnotes	Field notes taken by both facilitators in word format

### Data analysis

We compiled all collected data into a Microsoft Word document and imported it into the qualitative data analysis software MaxQDA (VERBI.GmbH 2021). The database consisted of three sections: the preparation activity, the first co-design session, and the second co-design session. The different data types were combined: field notes, image documentations of the mind maps and boards showing sticky notes and comments. We also listened to and transcribed part of the workshop recordings to validate and enrich our data. We organised this document using headings and categorised data according to the activities within each section. Two researchers then analysed the collected qualitative data using a reflexive and recursive approach to thematic analysis, which involved: 1) familiarisation, 2) generating codes inductively and deductively, 3) constructing themes, 4) revising and defining themes, and 5) producing a report (Braun and Clarke 2021).

## Findings

The analysis showed that the co-design activities stimulated reflection and engagement with the ethical principles, with five key themes emergent: 1) clarity/unclearness, 2) operationalisation, 3) stakeholders, 4) impacts of not upholding principles, 5) interconnectedness. Concurrently, the co-design activities brought about reflection on how visuals could help and hinder the communication of ethical principles.

### Reflection on the ethical principles

#### Clarity/unclearness

Reflection on the meaning of principles revealed first a need for clarity in the descriptions and terms used. In the show & tell exercise, one participant illustrated the challenge of grasping ethical concepts using the visual metaphor of a fish tank. She used the solid fish and corals to represent the clarity of data processing activities, whilst the water signified ethics. She also remarked that *“you have to read the guidelines like five times to make sure you get all the points*.” In the scenario-building activity, participants reiterated that some terms describing the principles were too vague and needed further clarification. For example, in relation to both Data Fairness and Accountability, participants asked, “*what even is fair”* and *“fair to whom?”* They also speculated whether the principle of Data Fairness had the same meaning as Fair Data (Findable Accessible Interoperable Reusable) (Reiser et al. 2018).

#### Operationalisation

Participants continuously expressed a desire for more operationalisation of the ethical principles. One participant reflected, “*for me, the gap is between the guidelines and the practical recommendation. What does it mean as a researcher? And how to implement that*?” Similarly, another commented – “*I did not really know what to look for, what to do with it (the framework)*” and represented this using a figure scratching its head. The desire for operationalisation continued as a theme in the scenario building and discussions. For example, participants highlighted difficulties in implementing Privacy due to data types’ diverging requirements and privacy standards, alongside the lack of standardised terminology. Illustrating this was a research collaboration story where different terms complicated compliance, as the hospital used the term ‘medical data’ for which the institute used ‘health-related data.’ Despite such challenges, participants described Privacy as the most operationalisable principle as they often had to write about it in their ethical approval processes. This suggests the instrumental role of ethics committee reviews in sensitising researchers’ awareness and understanding of ethical principles.

#### Different stakeholders

The co-design activities resulted in the identification of the many stakeholders involved and impacted by data sharing within a health research context. Particularly in the scenario-building activity, participants identified health providers, research institutes, scientists, data managers, data harvesters (such as Google), private companies, hackers, journals, media, patients, and broader communities. The discussion moved from their different perspectives to their responsibilities, alongside the challenges of finding a balance between expectations. For example, in one scenario, a participant told of her first-hand experience juggling the diverging requirements for data processing and sharing of the research institute compared with the collaborating hospital. By sharing their personal experiences, participants started to connect ethical principles to real-world examples, allowing them to see their relation and relevance.

#### Impacts of not upholding principles

Participants also reflected on the consequences of disrespecting the ethical principles. Expected consequences ranged from damaging media attention, lawsuits and negative impacts on patients and the public, such as stigmatisation, decreasing trust or privacy due to insufficient data security and unauthorised data accessing. Harms to research were also mentioned, such as the increased need for time and resources, challenging collaborations, and ultimately unclear resolutions that impede research. One participant also noted that undermining Respect of Persons could lead to de-personalisation of participants and forgetting the wider context, as “*when you work with numbers and databases you start seeing people only as organ donors*.” In this sense, participants highlighted the significant impact of terminology describing stakeholder roles, specifically that of study participants. For example, a participant explained that calling participants ‘data subjects’ could reduce them to their functionality in a study, while calling them ‘patients’ would imply a health condition.

#### Interconnectedness

Finally, reflection on the ethical principles revealed their interconnectedness, with participants frequently discussing how the principles related to each other. One participant, for instance, considered Respect for Persons as the overarching principle, arguing it is the foundation for all other principles. Interconnectedness was also evinced through the scenario-building exercise, wherein groups used the same scenario to illustrate different principles. For example, they used the Covid-19 Vaccination Information leakage case in Switzerland (Fichter and Seemann 2021), to demonstrate the need for respecting all four principles.

### Reflection on visualisation

The representation of the ethical principles also emerged as a key result of the co-design process. Even without instruction, participants approached making sense of the principles through imagery. “*It draws a picture in my mind”* remarked one participant*.* Data analysis further revealed that participants generally believed visuals could improve and motivate engagement with ethical frameworks. One participant commented, “*the guideline was very informative… but very dry to read*’ and signified this with an image of rice. Participants subsequently suggested visuals could promote engagement, understanding and memorisation by distilling the information, making abstract concepts more concrete, and providing potential structure to the document. Participants also reflected that visuals could do harm. For instance, visuals could convey the wrong message by oversimplifying the content, not telling the whole story, or causing suspicion when branding is inappropriate (for example, using a pharma industry logo). As well, they identified the challenge of visually representing diversity and ensuring inclusion. One participant reflected that she understood how difficult it is to visually include all stakeholders, saying “*it’s hard to address all the aspects of diversity.*” However, using a simplistic approach (for example, a non-gendered image) was also identified as exclusive, with one participant sharing that she did not relate to the icon of a male, able-bodied form often used to indicate a person.

Participants then suggested various ways to address the potential shortcomings of visuals. Recommendations included using lay language, showing the perspective of both the researcher and study participant, and ensuring that audiences can easily interpret the visual. The voting exercise captured participants’ ideal design criteria (see supplementary file 1), and through group discussion agreement was reached as to what would be tested in sketching and prototyping. The following sketching and ideation process then resulted in an array of referents and ideas to communicate each principle. Respect of Persons was paired with images of hands to represent solidarity and human figures for human dignity. Privacy was represented with commonly used symbols, such as images of shields and locks to show protection, alongside a masked person to signify anonymity. Accountability was translated through law symbols, such as an official stamp to show quality, and a signature to indicate someone responsible. Data Fairness was conveyed using a scale for fairness, a server or paper records to show data, and the metaphor of a cake to convey sharing. Interestingly, participants found it challenging to visually represent data, as while some saw data in a computerised, digital form, others imagined data as stacks of paper with pie charts and analysis.

### Feedback on the online workshop format

In complementation to reflections and discussion within the workshop, six out of the eight participants completed voluntary workshop evaluation questionnaires. One survey response was excluded from the analysis as it was incomplete. All completed responses (5/5) found the workshop met expectations, was interesting, enjoyable, useful, and stimulated reflection on both the ethical principles alongside the role of visual communication. In an open comment, one participant wrote, *“I have them (ethics principles) more in mind for the day’s work. It was a refreshment or reminder of the pillars of our research.”* Whilst another reported*, “they feel less scary…”.* Similarly, 5/5 reported having a good experience collaborating, and 4/5 felt the workshop succeeded in exploring how visuals could help or hinder communication. As to the activities themselves, the majority of responding participants enjoyed the sketching and voting activities most (4/5). The below anonymous comment demonstrates that although the online format was not ideal for all activities, namely sketching, the overall experience was still positive, providing both skills and learning.

“In-person meeting would have been better, especially for the sketching and for encouraging participation in the discussions. During in-person meetings, even if not everyone talks, it is possible to read people´s faces, but in Zoom with no camera (switched on) that gets lost. Saying that, you did a good job reminding participants to talk and giving positive reinforcement for people´s contributions. Overall, I'm really happy I participated, and I take home new skills to apply to my professional and personal life. I look forward to see the final design.” – Anonymously commented by one participant

### Visualisation outputs

Participation post-workshop took various forms. Following the INVVOLVE framework (Slattery et al. 2020), we gave co-design participants control and choice by asking them how they would like to be further involved in the project. One participant wanted to work as a designer, five participants chose the role of decision-maker, and four participants decided for no further involvement. The participant in the role of the designer, together with the principal researcher, drafted four visuals. Decision-makers then used five criteria identified from the workshops to evaluate the drafted designs. These criteria are outlined below. In an iterative process, visuals were created of the four principles Respect of Persons, Privacy, Accountability and Data Fairness. A knowledge visualisation expert also provided feedback based on these design criteria, with all resulting changes approved by the workshop participants.

Criteria guiding the decision makers’ and expert’s feedback.

- The visual is easy to interpret
- The visual conveys the right message
- The visual makes the concept more concrete
- The visual does not oversimplify the concept
- The visual represents diversity and inclusion
- The visual considers both the perspective of the researcher and researcher participant

**Fig. 2:**
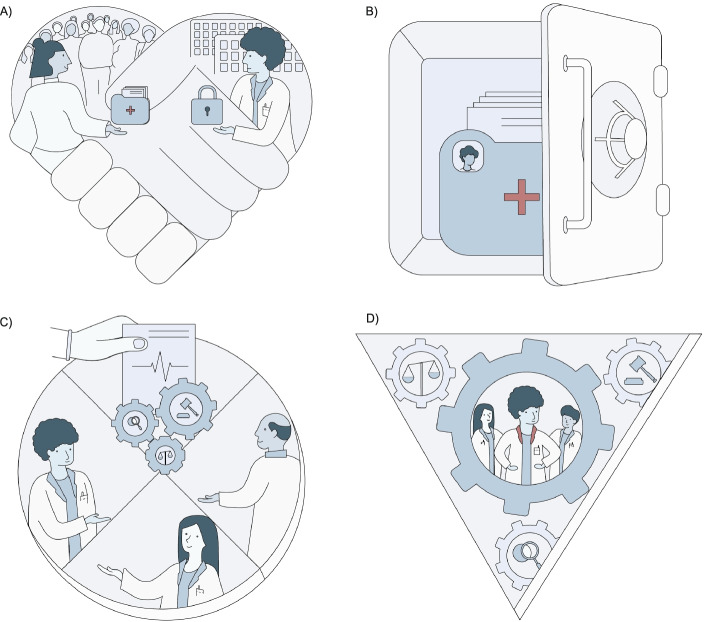
Final visualisations of the ethical principles: **a**) Respect of persons, **b**) Privacy, **c**) Data fairness, **d**) Accountability

As Fig. 2 shows, each resulting visual has its’ own unique shape aiming to aid visual memory encoding and retrieval. Perspective and shading draw the viewer’s attention to what’s inside the shapes, alluding to how the principles help frame issues. SPHN’s branding, along with the design criteria voted on during the workshops, determined the visual style, use of curved lines and the colour palette. Regarding the referents, participants decided on using metaphor alongside visually observable phenomena and icons. For Respect of Persons, heart-shaped hands embrace to visually signify an equal and respectful relationship between participants, researchers, and their institutes. Participant diversity gets conveyed by a crowd, and the need for researchers to respect participant’s privacy and confidentiality is shown by a lock. To convey the principle of Privacy, participants tossed up between imagery of locks and shields. In the end, they agreed on a vault to emphasise that privacy was linked to health data storage, access and sharing. For the principle of Accountability, participants agreed to graphically represent a person accountable. Icons surrounding this person then show the qualities of being fair, lawful, transparent, and are placed in cogwheels to indicate their relation to accountability mechanisms. Participants discussed at length how to visualise the principle of Data Fairness due to the difficulty of representing the broadness of data and data processing concepts. Finally, the group decided to communicate the principle through the metaphor of a data cake, an idea that emerged during the second workshop. In this way, the visual highlights how data fairness is primarily about sharing data. It is important to note that these visuals were not designed to function as stand-alone, rather they were designed to be positioned within the SPHN framework and read alongside the text to promote multi-modal communication.

## Discussion

This study explored whether engaging early-stage health researchers in visualising ethical principles could foster engagement and reflection on these principles and therefore bring ethics down from the heavens. This supports Steen’s (2013) conception of co-design, informed by the ideas of philosopher John Dewey, as a “*process of joint inquiry and imagination… (for) when people engage in a co-design process, they also engage in ethics*.” Aristotle embodies this in the concept of ‘technē’ meaning knowing by making’ (Wang 2013), just as Kleinsmann and Valkenburg’s (2008) describe co-design as different actors sharing their knowledge about ‘*both the design process and the design content… to create a shared understanding… and to achieve the larger common objective: the new product to be designed.*’ The study findings echo this, showing how the co-design activities resulted in engagement and reflection on ethical principles, alongside the development of design outputs, visuals which now represent a new resource for health research ethics education. Furthermore, this pilot study provides several insights relevant to ethics educators interested in applying co-design approaches within online environments. To support educators with such endeavours, we report below several key learnings and recommendations.

### Key learnings

#### Logistics of technology-mediated workshops with design

Conducting the co-design workshops in an online environment had many benefits and logistical hurdles. For one, the online setting provided the opportunity to include participants from different locations. For example, we had participants from different countries, who would otherwise remain physically separated. However, to accommodate for this broader geographical inclusion we needed a timeslot that fit two time zones, a restriction which resulted in some participants being unable to attend. In transforming this workshop to an online setting using Zoom we also considered the phenomenon of ‘Zoom fatigue,’ referring to the negative experience of extended computer mediated communication as normalized in the pandemic (Nadler 2020). To accommodate for this issue, we reduced workshop times from an entire day (which is usual for such workshops) and included short breaks after each activity, alongside a diversity of participation formats. Positive feedback from our participants suggests the issue of Zoom fatigue was averted. Educators and those supervising junior researchers interested in applying the activities and methods in online environments should thus consider time management, inclusion of breaks and diversity of activity formats.

#### Expanding design spaces

Another interesting hurdle in the digitalisation of the study’s activities was the question of how to conduct sketching and ideation in an online setting? Rather than working in one room and being able to make doodles and sketches, participants in our workshops remained in their home environments with the instruction to sketch on their paper and then show this to the group on their computer screen. This approach worked for most participants, with some participants going even a step further and using self-selected tools and programs to sketch their ideas digitally. However, some participants instead chose to document their ideas only using post-it notes. This experience showed us the importance of being open to adapting procedures and tools for co-design by giving participants freedom of choice in their medium of expression. This study thus shows how ethics educators can apply visualisation exercises and activities (such as sketching) even in online settings when distance learning is required.

#### Using visuality to explore ethical concepts

The study findings show the inherent challenge of pinning down the meaning of ethical principles even during reflection and deliberation processes. We see this in the analysis, which indicates that whilst most participants enjoyed the experience, they found it challenging to agree on the exact meaning and operationalisation of abstract and contested concepts, such as fairness. This result echoes the European Commissions’ (Guidelines 2017) Report of the Working Group on mHealth Assessment Guidelines in which “*a minimal level of consensus between the members of the Working Group was not reached*,” which rendered it “*impossible to achieve and endorse any guidelines*.” However, certain concepts, such as fairness, have inherent ambiguity, which also has its advantages, for this elasticity allows for the inclusion of a range of strategies, operationalisations, and tactics across domains (Hoffmann et al. 2018). Mittelstadt (2019) expands on this by stating that “*ethics is not meant to be easy or formulaic. Intractable principled disagreements should be expected and welcomed, as they reflect both serious ethical consideration and diversity of thought*.” Scholars warn against oversimplification as “*a temptation to which moral philosophers are not immun*e” (Toulmin 1981). Interestingly, the process of visualisation did resolve this issue in that it helped participants conceptualize the principles and their related concepts. For example, with the principle of Privacy the visualisation process ignited a deeper discussion. Only when visualizing Privacy with a lock and a shield did participants reflect that in this framework the concept referred to more than just data protection, to also embody the secure storage and sharing of data and samples. They then chose to convey this visually using a vault.

#### Incentivise participation for larger, more diverse samples

The limited budget and voluntary participation in the study resulted in only a small sample of early-stage researchers. Furthermore, our participants were a relatively homogenous group of early-stage researchers who had not worked on SPHN projects and were interested in design. Although we tried to diversify the group with more senior researchers, they reported to all be time-poor and overcommitted, rendering their participation impossible. Therefore, the sample does not reflect the diversity of the SPHN framework users, and the results do not allow for generalisable conclusions. One recommendation for future studies is thus the addition of incentivisation mechanisms to reduce participation drop-outs and help ensure a larger sample is obtained.

#### Time-management and evaluation measures

Co-design workshops usually take place in person and can span various days. Our need for a digital approach and the pandemic context limited in-person activities, whilst the time restriction in the online workshop may have hindered the depth of discussion. A further challenge was that only six participants filled out the online evaluation survey which we distributed after the workshops to save time for collective activities. To resolve this, we recommend future studies schedule time within the workshop for evaluation measures. Future research could use a pre and post workshop assessment design to validate co-design as an approach that fosters engagement with and reflection on ethical principles in health data research, including in the educational settings (i.e., PhD programs).

## Conclusion

The results of this explorative pilot study highlight that the co-design process is more than just a means to an end (i.e., designing engaging visuals), as it allows for in-depth engagement and deliberation about the meaning of ethical principles and their relevance to health research. Further, our findings suggest that engaging researchers in a co-design process visualising ethical principles appeals to their creativity and simulates reflection far beyond an ethics checklist approach. Highlighting the benefits and feasibility of co-creation formats, our participants perceived these design-oriented, creative activities as enjoyable and to have brought some diversion to their usual routine and working style. While the findings of this pilot are not generalisable, the results do provide the foundations for future research to validate co-design and visualisation approaches as educational and reflective tools for doctoral and postdoctoral training programs in the health sciences.

## Supplementary Information

Below is the link to the electronic supplementary material.

Supplementary file1 (PDF 220 KB)

## Data Availability

Data supporting study findings are available within the article and supplementary materials.

## Abbreviations

SPHN: Swiss Personalised Health Network
