# Supplementary material for: Fostering ethical reflection on health data research through co-design: A pilot study
Source: Int J Ethics Educ. 2022 Jun 22;7(2):325–42. doi: 10.1007/s40889-022-00148-4 (PMC9214681; doi:10.1007/s40889-022-00148-4)
Supplement: Supplementary file 1 — Supplementary file1 (PDF 220 KB) [file 40889_2022_148_MOESM1_ESM.pdf]

S1. Screenshot of mind map activity

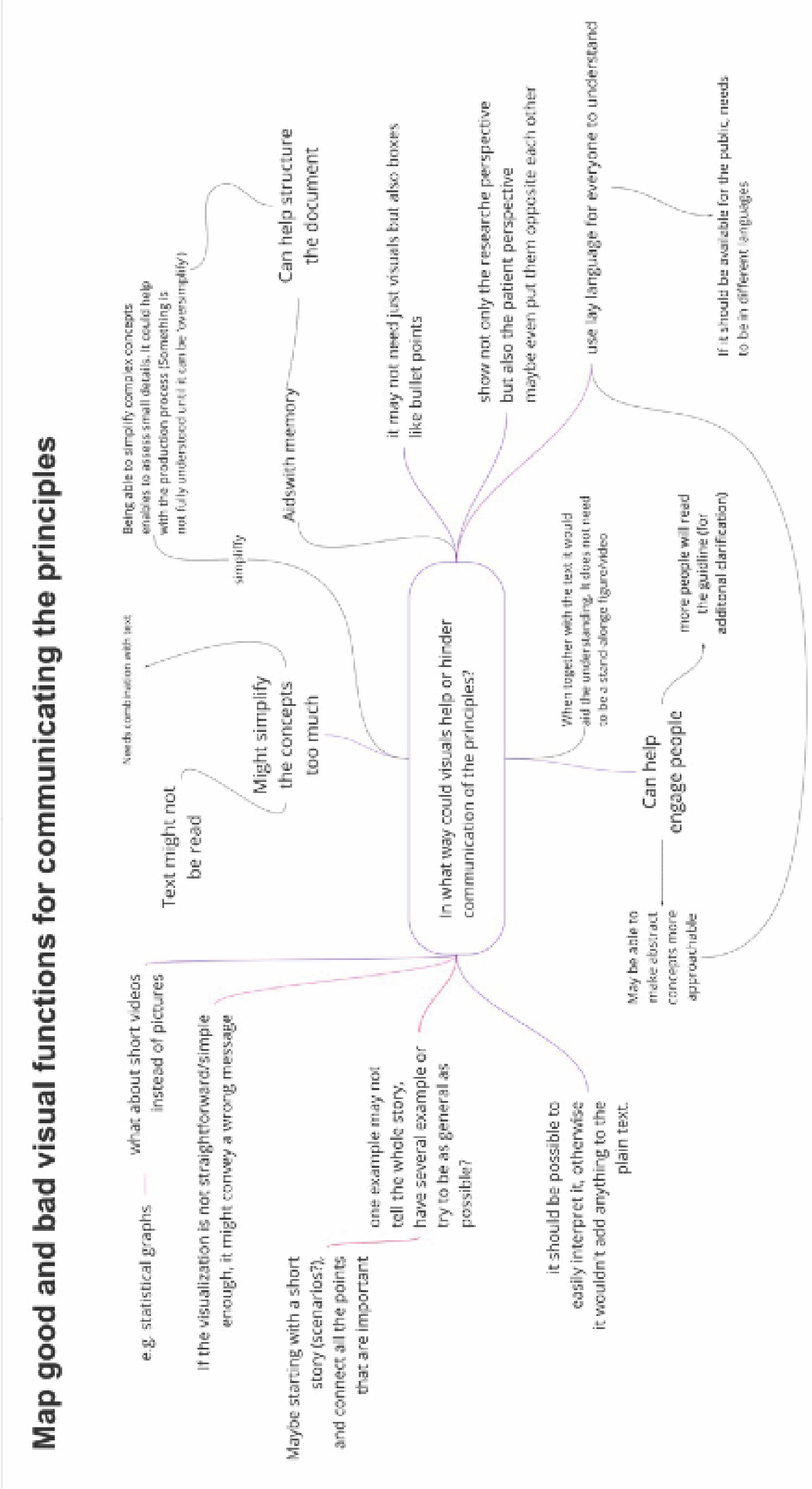

S2. Results from design criteria voting

Group	Item	Votes
Graphic Type	Illustration	3
	Icons or Symbols	4
	3D model	2
	Photograph	0
	Data Viz.	1
	Diagram	4
Engagement	Static	3
	Animated	1
	Interactive	2
Detail	Simple	4
	Details	0
Representational Convention	Realistic	0
	Schematic	4
	Metaphor	5
Story	Narrative	4
Colour	No colour	0
	Maybe colour	2
	Yes Colour	3
Text	No text	0
	Maybe some text	5
	Yes text	1
Style	Minimal / simple	3
	Professional / corporate	3
	Hand drawn	1
	Playful	3
	Comic / Cartoon	2
	Collage	0
